# Polymorphisms of CLEC16A Region and Autoimmune Thyroid Diseases

**DOI:** 10.1534/g3.114.010926

**Published:** 2014-03-18

**Authors:** Fatuma-Said Muhali, Tian-tian Cai, Jiao-li Zhu, Qiu Qin, Jian Xu, Shuang-tao He, Xiao-hong Shi, Wen-juan Jiang, Ling Xiao, Dan-Feng Li, Jin-an Zhang

**Affiliations:** * Clinical Research Center, The First Affiliated Hospital of Medical School of Xi’an Jiaotong University, Xi’an, People’s Republic of China, 710061; † Department of Endocrinology, Jinshan Hospital of Fudan University, Shanghai, People’s Republic of China, 201508

**Keywords:** CLEC16A, single-nucleotide polymorphisms (SNPs), autoimmune thyroid diseases (AITDs), Graves’ disease (GD), Hashimoto’s thyroiditis (HT), innate immunity, complex genetics, tolerance, complex immunity, infection, resistance

## Abstract

To investigate the association of CLEC16A gene polymorphisms and autoimmune thyroid diseases (AITDs). Six hundred sixty seven Han Chinese patients with AITDs were selected as study subjects, including 417 patients with Graves’ disease (GD), 250 patients with Hashimoto’s thyroiditis (HT) and 301 healthy control patients. Polymerase chain reaction-restriction fragment length polymorphism (RFLP) and the mass spectrometry technique were used to genotype five CLEC16A single-nucleotide polymorphisms (SNPs) (rs12708716, rs12917716, rs12931878, rs2903692, and rs6498169). Higher frequency of G allele of rs6498169 CLEC16A gene in AITDs patients [*P* = 0.029, odds ratio (OR) 1.29 and 95% confidence interval 1.022−1.505] was observed. In addition an association between rs6498169 and HT was observed with statistical significance (*P* = 0.018, OR 1.335, 95% confidence interval 1.051−1.696). Furthermore, the GG haplotype containing the major allele of (rs12708716 and rs6498169) was associated with an increased risk of HT (*P* = 0.0148, OR 1.344). When patients with HT and controls were compared, results from the dominant and recessive models showed that the genotype frequency of rs6498169 were at borderline levels (*P* = 0.054 and *P* = 0.05), and the other four SNPs of CLEC16A gene showed no significant association with AITDs. Our results suggest that polymorphisms rs6498169 of CLEC16A gene confers susceptibility to AITDs. We therefore disclose for the first time the association of rs6498169 SNP with AITDs.

Autoimmune thyroid diseases (AITDs) are archetypes of organ-specific diseases, where disease phenotypes seem to derive from the interaction of genetic and environmental factors. AITDs mainly consist of Graves’ disease (GD) and Hashimoto’s thyroiditis (HT). It is known that genetic factors play an important role in the development of AITDs. A number of twin studies show approximately 75% of the total phenotypic variance in AITDs is because of genetic effects (Brix and Hegedüs 2012). AITDs genetic etiology is well established but the main genetic variants and their epistasis still need to be uncovered. Specific genes and alleles are necessary, such as human leukocyte antigen (HLA) (Ban *et al.* 2002; Simmonds *et al.* 2005), cytotoxic T lymphocyte-associated antigen (CTLA-4) (Kavvoura *et al.* 2007), protein tyrosine phosphatase-22 (PTPN22) (Heward *et al.* 2007), and thyroid-stimulating hormone receptor gene (TSHR) (Tonacchera and Pinchera 2000; Maierhaba *et al.* 2008) but they are not sufficient for disease development. In order to identify additional risk variants, we examined the distribution of the allele and genotypes of CLEC16A gene in AITDs patients.

CLEC16A (C-type lectin domain 16, previously known as KIAA0350) is located in a 233-kb linkage disequilibrium block on chromosome 16p13. CLEC16A is a protein in humans that is encoded by the CLEC16A gene. It is a member of the C type lectin family. Members of this family are thought to provide an important signal for immune tolerance (Davison *et al.* 2012). However, this protein lacks crucial domains in carbohydrate recognition, which can be involved in diverse processes, including plasma glycoprotein turnover, cell–cell adhesion, and innate pathogen recognition. Until now, little is known about the detailed function of this protein, despite it being highly expressed on B-lymphocytes, natural killer (NK) and dendritic cells (DC) (Skinningsrud *et al.* 2008).

Up to now, a number of studies showed significant associations of CLEC16A gene with autoimmune diseases such as type 1 diabetes (Cooper *et al.* 2009; Sang *et al.* 2012), multiple sclerosis (International Multiple Sclerosis Genetics Consortium 2009; Zoledziewska *et al.* 2009), and juvenile idiopathic arthritis (Skinningsrud *et al.* 2010). To better understand the role of CLEC16A as an autoimmune risk locus and the association between CLEC16A and AITDs, this research was performed.

## Materials and Methods

### Experimental subjects

All AITDs (GD and HT) patients and controls in the present case-control study were Chinese Han population. They were recruited in the endocrinology outpatient department of First Affiliated Hospital of Xi’an Jiaotong University. The research project was approved by the ethics committee of the hospital. We investigated 667 non-related AITDs patients consisting of 417 GD patients (124 males and 293 females; ages 5–73 years 34.48 ± 13.95 years) and 250 HT patients (39 males and 211 females; ages 4–77 years, 31.90 ± 13.10 years). The detailed information of the subjects was shown in Table 1. The diagnostic criteria for AITDs were based on our previous published paper (Maierhaba *et al.* 2008). GD patients were diagnosed by clinical manifestations and biochemical assessments of hyperthyroidism and the positive circulating thyroid-stimulating hormone receptor antibody (TRAb), with or without positive antibody against thyroid peroxidase (TPOAb) or antibody against thyroglobulin (TGAb) and diffuse goiter of the thyroid. HT was defined based on the high level of either TPOAb or TGAb, with or without clinical and biochemical hypothyroidism and the presence of an enlarged thyroid. And 301 healthy subjects (91 males and 210 females; ages 10–72 years, 33.60 ±12.64 years) without clinical evidence (goiter, TPOAb, and TGAb) and family history of any autoimmune diseases were selected from the health care center of the same hospital and were used as controls.

**Table 1:** Clinical data of AITDs patients and controls

	GD	HT	Control
N	417	250	301
Sex			
Male	124 (29.74%)	39 (15.60%)	91 (30.23)
Female	293 (70.26%)	211 (84.40%)	210 (69.77%)
Age	34.48 ± 13.95	31.90 ± 13.10	33.60 ± 12.64
Onset of age	32.31 ± 14.07	30.29 ± 13.05	
Thyroid size			
Normal	55 (13.19%)	21 (8.40%)	
First degree	65 (15.59%)	37 (14.80%)	
Second degree	231 (55.39%)	169 (67.60%)	
Third degree	66 (15.83%)	23 (9.20%)	
Family history			
(+)	72 (17.27%)	54 (21.60%)	
(−)	345 (82.73)	196 (78.40%)	
Ophthalmopathy			
(+)	98 (23.50%)	6 (2.40%)	
(−)	319 (76.50%)	244 (97.60%)	

AITDs, autoimmune thyroid diseases; GD, Graves’ disease; HT, Hashimoto’s thyroiditis.

### Genotyping

1−2 mL peripheral venous blood was collected from all subjects. The genomic DNA was extracted from the blood by using RelaxGene Blood DNA System (Tiangen Biotech, Beijing, China). Marker-tagging single-nucleotide polymorphisms (SNPs) were selected from the Hapmap CHB data using the Haploview software to meet the following criteria: minor allele frequency >0.1 and Hardy-Weinberg equilibrium (HWE) with *P* > 0.001. rs12708716, rs12917716, rs12931878, rs290369, and rs6498169 SNPs were typed by a matrix-assisted laser desorption ionization-time of flight mass spectrometer (MALDI-TOF-MS) platform from Sequenom (San Diego, CA). Primer sequences for the targeted loci are as follows.

rs12708716 Forward: ACGTTGGATGACTTCATCCTCACTGACTCC

Reverse: ACGTTGGATGTCTCGGGTCTTCAGCTAGTC

rs12917716 Forward: ACGTTGGATGGCTTTTAGAGCAAGAACCAG

Reverse: ACGTTGGATGCTGCTATGAGGGCCTTTCCA

rs12931878 Forward: ACGTTGGATGCAGAGACCACAGGTATGAAG

Reverse: ACGTTGGATGAACACTCCCAGCTTGCAAAC

rs2903692 Forward: ACGTTGGATGCTAGGTCTGGTACAAAAGTG

Reverse: ACGTTGGATGTGGGAAATGGGTTCTGGATG

rs6498169 Forward: ACGTTGGATGTGTGGGACATGGCTAACCG

Reverse: ACGTTGGATGCTAAAGCCAAGTTGCTGCTC

### Statistical analysis

The clinical data were described as mean± SD (mean ± standard deviation). Allele and genotype frequencies between cases and controls were compared using the chi-square test. The odds ratio (OR) and 95% confidence interval (95% CI) were calculated. All statistical analyses were performed using the software SPSS, version 16.0. And *P* < 0.05 was considered as statistically significant. Haplotypes for SNPs were tested for Hardy-Weinberg equilibrium using Haploview 4.0 analysis platform. Some investigators use multiple tests to examine multiple candidate cut-points and adjust their *P* values. All *P* values presented in this study were uncorrected.

### Clinical phenotype analysis

Clinical data of AITDs patients and control groups were analyzed in the following ways: Clinical manifestations at the time of recruitment was divided into five parts: (1) gender: the number of male and female patients; (2) age of onset: (data are expressed as mean ± SD); (3) thyroid size: the size is divided into three degrees by palpation: first degree, the goiter cannot be seen but can be palpated; second degree, the goiter can be seen and palpated, but within the sternocleidomastoid; third degree, the goiter exceeds the exterior margin of sternocleidomastoid; (4) family history: the incidence of a disease of the patient’s first and second degree family members; (5) ophthalmopathy, defined as distinctive disorder characterized by inflammation and swelling of the extraocular muscles, eyelid retraction, periorbital edema, episcleral vascular injection, conjunctival swelling, and proptosis.

## Results

### Allele and genotyping results

For rs6498169, the G allele was significantly more frequent in patients with AITDs (*P* = 0.029, OR 1.29, 95% CI 1.022−1.505) specifically in patients with HT (*P* = 0.018, OR 1.335, 95% CI 1.051−1.696) than in controls, but no association of this allele and GD was observed. Furthermore, we did not find any significant difference between patients and controls in all the other four SNPs (results shown in Table 2).

**Table 2:** Allele distribution of CLEC16A gene in patients with AITDs

SNP Name	Allele/Genotype	Control (%)	AITD (%)	*P* Value	GD (%)	*P* Value	HT (%)	*P* Value
rs12708716	A	451 (74.92)	1016 (76.16)	.554	633 (75.90)	.669	383 (76.60)	.517
	G	151 (25.08)	318 (23.84)		201 (24.10)		117 (23.40)	
rs12917716	C	244 (40.53)	504 (38.24)	.339	312 (37.77)	.291	192 (39.02)	.612
	G	358 (59.47)	814 (61.76)		514 (62.23)		300 (60.98)	
rs12931878	A	516 (86.0)	1167 (87.48)	.370	727 (87.17)	.52	440 (88.00)	.327
	G	84 (14.0)	167 (12.52)		107 (12.83)		60 (12.00)	
rs2903692	A	156 (25.91)	303 (22.71)	.125	192 (23.02)	.207	111 (22.20)	.152
	G	446 (74.09)	1031 (77.29)		642 (76.98)		389 (77.80)	
rs6498169	A	290 (48.33)	571 (43.0)	.029	365 (44.08)	.112	206 (41.20)	.018
	G	310 (51.67)	757 (57.0)		463 (55.92)		294 (58.80)	

AITDs, autoimmune thyroid diseases.

Table 3 shows genotype distributions of rs12708716, rs12917716, rs12931878, rs2903692, and rs6498169 in patients with AITDs and controls. rs6498169 showed a marginally significant trend between HT subjects and control group under codominant model (*P* = 0.058), dominant model (*P* = 0.054) and under recessive model (*P* = 0.050). The GG genotype was higher in patients with HT, which indicates that the GG genotype could increase the susceptibility to HT. We failed to detect the genotype of few patients, both in rs12917716 and rs6498169, and these patients were excluded from the statistical analysis.

**Table 3:** Genotype of the CECL16A SNPs in patients with GD and HT and controls

SNP	Genotype	Control (%)	GD (%)	GD *vs.* Controls (*P*)			HT (%)	HT *vs.* Controls (P)		
				Codominant	Dominant	Recessive		Codominant	Dominant	Recessive
rs12708716	AA	167 (55.48)	243 (58.27)	0.590	0.456	0.648	148 (59.20)	0.616	0.380	0.860
	AG	17 (5.65)	27 (6.47)				15 (6.00)			
	GG	117 (38.87)	147 (35.25)				87 (34.80)			
rs12917716	CC	48 (15.95)	66 (15.98)	0.274	0.990	0.131	41 (16.67)	0.569	0.820	0.367
	CG	105 (34.88)	167 (40.44)				95 (38.62)			
	GG	148 (49.17)	180 (43.58)				110 (44.72)			
rs12931878	AA	222 (74.00)	317 (76.02)	0.814	0.537	0.750	193 (77.20)	0.584	0.385	0.462
	AG	6 (2.00)	7 (1.68)				3 (1.20)			
	GG	72 (24.00)	93 (22.30)				54 (21.60)			
rs2903692	AA	19 (6.31)	26 (6.24)	0.282	0.966	0.127	15 (6.00)	0.227	0.879	0.092
	AG	164 (54.49)	251 (60.19)				154 (61.60)			
	GG	118 (39.20)	140 (33.57)				81 (32.40)			
rs6498169	AA	69 (23.00)	83 (20.05)	0.251	0.342	0.109	41 (16.40)	0.058	0.054	0.050
	AG	79 (26.33)	132 (31.88)				85 (34.00)			
	GG	152 (50.67)	199 (48.07)				124 (49.60)			

SNP, single-nucleotide polymorphism; GD, Graves’ disease; HT, Hashimoto’s thyroiditis.

### Genotype, allele distribution, and clinical phenotype association in AITDs patients

Patients with AITDs were divided into family history-positive and family history-negative, and genotype and allele distribution were compared in all the five SNPs; unfortunately, no significant association was observed. The patients were also grouped according to the size of the thyroid, that is, those with third degree compared with those with first and second degree. The results also were not significant (data not shown).

### Haplotype analysis

**Figure 1:**
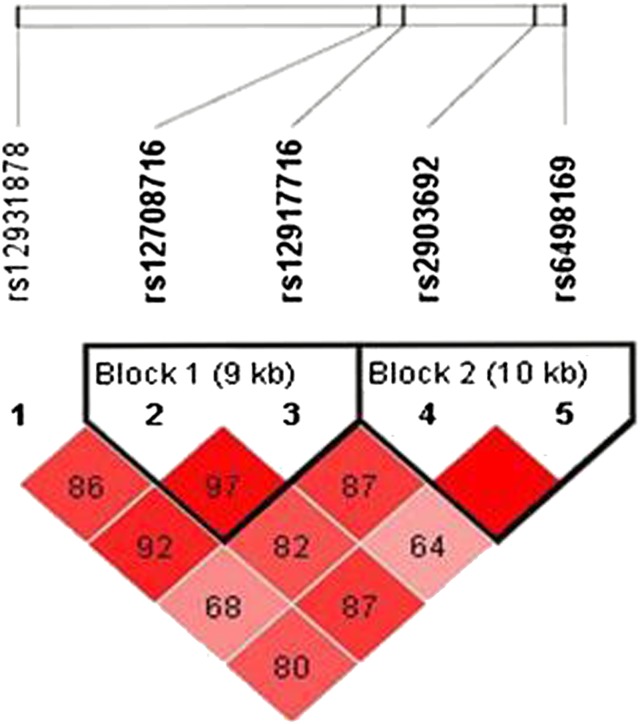
Linkage disequilibrium (LD) block defined by the Haploview 4.1.

Table 4 shows the frequency of the GG haplotype in HT patient group was significantly higher than that in control group (*P* = 0.015, OR 1.344). It implies that GG was the risk haplotype to HT. However, the haplotypes of block 1 were neither associated with GD nor HT. Two detected LD blocks are shown in Figure 1, rs12908716-rs12917716 and rs2903692-rs6498169, according to D′ value.

**Table 4:** Haplotype analysis in AITDs patients and controls

Haplotype	Control (Frequency)	GD (Frequency)	*P*	OR	95%CI	HT (Frequency)	*P*	OR	95% CI
Block1									
AG	355 (0.593)	43 (0.597))	0.928			303 (0.607)	0.658		
GC	150 (0.250)	18 (0.250)	1			115 (0.230)	0.44		
AC	94 (0.157)	11 (0.153)	0.932			81 (0.162)	0.821		
Block2									
GG	309 (0.515)	42 (0.583)	0.273			294 (0.588)	0.0148	1.344	1.058−1.708
AA	156 (0.260)	16 (0.222)	0.488			111 (0.222)	0.143		
GA	135 (0.225)	14 (0.194)	0.555			95 (0.190)	0.155		

AITDs, autoimmune thyroid diseases; GD, Graves’ disease; HT, Hashimoto’s thyroiditis; OR, odds ratio; CI, confidence interval.

## Discussion

CLEC16A gene is considered as a potential causal gene because it may encode an immunoreceptor tyrosine-based activation motif (ITAM), which can trigger properties of B-cell receptor (BCR), T-cell receptor (TCR), and Fc receptor (FcR) (Hakonarson *et al.* 2007). There are several SNPs in the CLEC16A gene linked with many autoimmune diseases. The gene was preliminarily tested for type I diabetes via genome-wide association study (GWAS) (Daëron *et al.* 1995), and this gene was later studied in various autoimmune diseases.

Some loci are involved in multiple autoimmune diseases, but the associated SNP and/or allele is not the same for each disease. For example, SNPs of TNFAIP3 region associated with systemic lupus erythematosus, rheumatoid arthritis, and type1 diabetes are different from SNPs associated with psoriasis and celiac disease (Plenge *et al.* 2007; Thomson *et al.* 2007; Nair *et al.* 2009). Similarly, our study differed in conclusion with Awata *et al.* (2009), who studied CLEC16A SNPs in type 1 diabetes complicated with AITDs and concluded that CLEC16A SNPs are not significantly associated with AITDs alone in Japanese. The findings from our study indicate the association between CLEC16A variant rs6498169 and AITDs (*P* = 0.029) and HT (*P* = 0.018). This maybe attributed to the fact that the risk loci for type 1 diabetes might be different from that of AITD. Many studies suggest that not only different populations may bring different relationships of gene studies and diseases but variant regions also can contribute to the different distribution of gene polymorphisms. Differences in ethnicity could also have been the reason for the variation in our results, but similar to Awata *et al.* (2009), our study failed to demonstrate a significant association between rs2903692 and AITDs. These results still require confirmation by other large-scale studies to get a better understanding of the contribution of the CECL16A gene to AITD.

We also showed that the G allele of rs6498169 of CLEC16A increased susceptibility to HT by 34% that is the result when rs6498169 SNP, G, and A alleles were compared in patients with HT and controls, *P* = 0.018 and OR 1.34 (not shown in Table 2). According to the research done by Johnson *et al.* (2010), there are some similarities in their conclusion where the G allele of rs6498169 has also showed increased in frequency in patients with multiple sclerosis. This finding indicates that many autoimmune diseases might share similar loci. Unfortunately, the genotype GG in rs6498169 only showed a marginally significant trend in HT.

Analyzing the correlation of the clinical subphenotypes and the alleles, none of the comparisons (cases with each clinical phenotype *vs.* those without) showed any statistical significance, likely because of the insufficient statistical power caused by the limited sample size after stratification. C-type lectin domain structure, which is expressed by dendritic cells, is crucial for tailoring immune responses to pathogens. Our results may add to the understanding of mechanisms for the genetic risk to AITDs as it is the complex disease with multifactorial pathophysiological mechanisms. As for now we don’t know the exact biological mechanism as to why CLEC16A gene is associated with HT and not GD, but possibly it reflects the different underlying pathogenic mechanisms of the two diseases. Whole-genome linkage studies on multiple families with AITDs have shown that multiple genes are responsible for a predisposition towards GD and HT and that some are common to both diseases and some are unique. Research on the exact mechanism of CLEC16A pathway on how it influences HT will be of interest to our future projects. It also should be noted that the lack of association of CLEC16A gene with GD in our study does not completely rule out CLEC16A as a candidate gene for GD. Studies with a larger sample size are required to further identify these SNPs carrying a smaller risk.

In conclusion, the CLEC16A gene is a newly discovered AITDs susceptibility gene. As interesting as the results are, still the G allele of CLEC16A is not explaining much about AITDs or HT. If these results are confirmed in other ethnic groups, they may help us understand something about the genetics of HT. Further effort is needed to understand the functional properties of CLEC16A, and more loci should be studied to uncover its association with AITDs.
